# Uremic Toxins Induce ET-1 Release by Human Proximal Tubule Cells, which Regulates Organic Cation Uptake Time-Dependently

**DOI:** 10.3390/cells4030234

**Published:** 2015-06-26

**Authors:** Carolien M. S. Schophuizen, Joost G. J. Hoenderop, Rosalinde Masereeuw, Lambert P. van den Heuvel

**Affiliations:** 1 Department of Pediatric Nephrology, Radboudumc 6525 GA Nijmegen, The Netherlands; E-Mails: carolien.schophuizen@gmail.com (C.M.S.S.); bert.vandenheuvel@radboudumc.nl (L.P.H.); 2 Department of Physiology, Radboud Institute for Molecular Life Sciences, Radboudumc 6525 GA Nijmegen, The Netherlands; E-Mail: joost.hoenderop@radboudumc.nl; 3 Department of Pharmacology and Toxicology, Radboud Institute for Molecular Life Sciences, Radboudumc 6525 GA Nijmegen, The Netherlands; 4 Division of Pharmacology, Utrecht Institute for Pharmaceutical Sciences, Faculty of Science, Utrecht University, Universiteitsweg 99 (room 2.72), 3584 CG Utrecht, The Netherlands; 5 Department of Pediatrics, Catholic University Leuven, 3000 Leuven, Belgium

**Keywords:** uremic toxins, endothelin signaling, cytokines, organic cation transport, iNOS, protein kinase C

## Abstract

In renal failure, the systemic accumulation of uremic waste products is strongly associated with the development of a chronic inflammatory state. Here, the effect of cationic uremic toxins on the release of inflammatory cytokines and endothelin-1 (ET-1) was investigated in conditionally immortalized proximal tubule epithelial cells (ciPTEC). Additionally, we examined the effects of ET-1 on the cellular uptake mediated by organic cation transporters (OCTs).

Exposure of ciPTEC to cationic uremic toxins initiated production of the inflammatory cytokines IL-6 (117 ± 3%, *p* < 0.001), IL-8 (122 ± 3%, *p* < 0.001), and ET-1 (134 ± 5%, *p* < 0.001). This was accompanied by a down-regulation of OCT mediated 4-(4-(dimethylamino)styryl)-*N*-methylpyridinium-iodide (ASP^+^) uptake in ciPTEC at 30 min (23 ± 4%, *p* < 0.001), which restored within 60 min of incubation. Exposure to ET-1 for 24 h increased the ASP^+^ uptake significantly (20 ± 5%, *p* < 0.001). These effects could be blocked by BQ-788, indicating activation of an ET-B-receptor-mediated signaling pathway. Downstream the receptor, iNOS inhibition by (N(G)‐monomethyl‐l‐arginine) l-NMMA acetate or aminoguanidine, as well as protein kinase C activation, ameliorated the short-term effects.

These results indicate that uremia results in the release of cytokines and ET-1 from human proximal tubule cells, *in vitro*. Furthermore, ET-1 exposure was found to regulate proximal tubular OCT transport activity in a differential, time-dependent, fashion.

## 1. Introduction

Renal transport processes are essential for the maintenance of body homeostasis. In the kidney, clearance of exogenous compounds and endogenous waste products from the circulation is facilitated by both glomerular filtration and active tubular secretion. The proximal tubule basolateral membrane transporters are responsible for the uptake of substrates from the circulation, while the apical transporters present in the proximal tubule facilitate their subsequent urinary release. In patients suffering from chronic kidney disease (CKD) or end-stage renal disease (ESRD), insufficient renal clearance and the subsequent accumulation of waste products lead to the development of uremia. Many solutes that accumulate in uremia have been identified [1]. However, we have only just begun to understand the effects that these compounds can exert on biological processes and their influence in the development of secondary morbidities. Previously, we demonstrated competitive inhibition of a selection of cationic uremic toxins (UTs) on organic cation uptake in a human conditionally immortalized proximal tubule epithelial cell model (ciPTEC) that endogenously expresses various renal transport proteins [2,3]. The clearance of cationic UTs (polyamines, guanidines and acrolein) largely depends on tubular secretion due to their high protein binding and/or compartmentalization [4,5]. In renal failure, the accumulation of these solutes is associated with inflammation, cardiovascular morbidity and perturbed erythropoiesis [6,7,8]. Moreover, conventional (hemo) dialysis methods are insufficient for their removal.

The development of a chronic inflammatory state is common in renal patients, and uremia has been identified as an important causative factor [9]. Increased levels of the vasoactive peptide endothelin-1 (ET-1), and pro-inflammatory cytokines such as IL-6, IL-8, and TNFα have been observed both before and after the start of dialysis therapy [10,11,12,13,14]. IL-6 promotes various inflammatory events, including the activation of lymphocytes, and is identified as a strong pro-fibrotic factor. Both IL-6 and IL-8 levels are correlated with increased mortality and poor disease outcome in renal failure [15]. TNFα is also known as an important factor in the development of renal fibrosis, and induces the production of additional inflammatory mediators such as ET-1 [16,17,18].

In healthy subjects, ET-1 functions as a potent peptide regulating the vascular tone, blood flow, and water and salt homeostasis. In the nephron, ET-1 mediates these processes through its tight regulatory effect on intracellular calcium, sodium and chloride channels, the production of phospho kinases and nitric oxide (NO) [19]. However, in patients suffering from renal disease, increased systemic ET-1 levels are also linked to a decline in renal function, the development of interstitial fibrosis, proteinuria, cardiomyopathy and glomerulosclerosis [20,21,22]. Our laboratory has previously demonstrated that exposure to nephrotoxicants can induce an ET-1-mediated signaling cascade in the proximal tubule. Upon stimulation, the proximal tubule produces ET-1 which can lead to activation of regulatory pathways including NOS and protein kinase C (PKC), ultimately resulting in a reduction of efflux transport mediated by two apical membrane transporters P-glycoprotein (P-gp) and multidrug resistance protein 2 (MRP2) [23,24,25]. On the other hand, long-term exposures resulted in an upregulation of the transport proteins, accompanied by nephroprotection [26,27].

In the present study, we investigated if a selection of cationic UTs, which were previously shown to interfere with tubular organic cation uptake, can act as nephrotoxicants and induce the local production of inflammatory mediators. Furthermore, we investigated if ET-1 can regulate the uptake of cationic compounds in human renal proximal tubule epithelial cells. To this end, the production of IL-6, IL-8, TNFα and ET-1 were assessed, and the short- and long-term effects of ET-1 on the regulation of organic cation influx transport in the human renal cell model were studied.

## 2. Experimental Section 

### 2.1. Chemicals

Spermine, spermidine, cadaverine, putrescine dihydrochloride, acrolein, guanidine hydrochloride, methylguanidine hydrochloride, and 4-(4-(dimethylamino)styryl)-N-methylpyridinium iodide (ASP^+^) were purchased from Invitrogen (Eugene, OR, USA). Tetrapentylammoniumchloride (TPA), insulin, transferrine, selenium, tri-iodothyronine, hydrocortisone, epidermal growth factor, Endothelin-1 (ET-1), BQ788, aminoguanidine (AG), and 8-Br-cGMP were purchased from Sigma-Aldrich Co. (Zwijndrecht, Netherlands). Sn-1,2-dioctanoyl glycerol (DOG) was obtained from Enzo Life-sciences (Raamsdonksveer, Netherlands). N(G)‐monomethyl‐l‐arginine (l-NMMA) acetate was purchased from Tocris bioscience (Bristol, UK)

### 2.2. Cell Culture

A previously developed and characterized immortalized human proximal tubule epithelial cell line, ciPTEC, obtained from a healthy volunteer urine samples [3,28] was cultured in Dulbecco’s modified eagle medium DMEM-HAM’s F12 (Lonza; Basel, Switzerland) containing 10% v/v fetal calf serum (FCS) (Greiner Bio-One; Alphen a/d Rijn, the Netherlands), 5 µg∙mL^−1^ insulin, 5 µg∙mL^−1^ transferrine, 5 ng∙mL^−1^ selenium, 36 ng∙mL^−1^ hydrocortisone, 10 ng∙mL^−1^ epidermal growth factor and 40 pg∙mL^−1^ tri-iodothyronine. CiPTEC were in culture for up to 40 passages, no antibiotics or phenol red was present during this time. Since the cell model was established after immortalization using hTERT and the temperature sensitive SV40t oncogenes, the cells proliferate at 33 °C and differentiate at 37 °C culture conditions [3]. Regular culture was performed at 33 °C 5% (v/v) CO_2_, media was refreshed every 2/3 days. For experiments, cells were seeded at a density of 1:3 and left to attach for 24 h at 33 °C. Subsequently, the cells were transferred to 37 °C to mature for 7 days prior to the experiments.

### 2.3. Enzyme-Linked Immuno Sorbent Assays

To quantify the production of IL-6, IL-8, and TNFα or ET-1 by ciPTEC under various culture conditions Enzyme-Linked Immuno Sorbent Assays (ELISAs) were performed. DuoSet® ELISA Development Systems; IL-6 #DY206, IL-8 #DY208, TNFα #DY210, ET-1 #DY1160 (R&D systems, Abingdon, UK) were used to accurately measure these compounds in complete culture medium supernatant. For all assays, 500 µL of media of exposed cells was harvested after the designated incubation period. The samples were centrifuged for 5 min at 7500 × *g* and the supernatant stored at −20 °C for a maximum of 2 months. For IL-6 and IL-8 the samples were diluted 500 times in phosphate buffered saline (PBS) or reagent diluent (PBS + 1% v/v FCS), respectively. The samples for TNFα and ET-1 were diluted 5× and 10× in PBS, respectively. The assays were subsequently performed according to the manufacturers’ protocol. The optical density of each well was measured immediately using the VictorX multilable plate reader (PerkinElmer, Waltham, MA, USA) set to 460 nm. To correct for optical imperfections in the plate, the readings at 540 nm were subtracted from these measurements.

Additionally, we tested the effect of a combination of cationic UTs on cytokines and ET-1 production. To mimic uremic conditions, a mixture of toxins was used comparable to 10 or 1 times the uremic plasma concentrations reported in literature; *viz.* spermidine 0.67 µM, spermine 0.09 µM, cadaverine 0.21 µM, putrescine 0.88 µM, acrolein 1.42 µM, guanidine 2.18 µM, and methylguanidine 7.66 µM [29,30,31,32].

### 2.4. qPCR

Total RNA was isolated using TRIzol (Life Technologies Europe BV, Zoetermeer, The Netherlands) and chloroform extraction according to the manufacturers’ protocol. 2 μg of total RNA served as a template for single-strand cDNA synthesis in a reaction using oligo (dT) and random primers in a M-MLV reverse transcriptase reaction mixture (Catalog #28025-013, Invitrogen, Bleiswijk, Netherlands) according to the manufacturers’ protocol (Doc. Rev: 100702). The mRNA expression levels were detected using gene specific primer-probe sets (Hs00174961_m1; Applied Biosystems, Foster City, CA, USA) and TaqMan Universal PCR Master Mix (Applied Biosystems). The CFX96 Real Time PCR system (Bio-Rad Laboratories, Veenendaal, Netherlands) was used to perform the qPCR reactions and data was analyzed using the CFX Manager^TM^ software (Bio-Rad Laboratories). The reference gene GAPDH was used to normalize the mRNA expression levels. Data are expressed as fold increase compared to proliferating ciPTEC.

### 2.5. OCT Mediated ASP^+^ Uptake

CiPTEC were cultured until confluence, in glass bottom Petri dish as described above. Cells were exposed to 100 nM ET-1 for 24 h, 30 min or taken as control. After washing the monolayer in Hepes Tris buffer (HT-Buffer: 132 mM NaCl, 4.2 mM KCl, 1 mM CaCl_2_, 1 mM MgCl_2_, 5.5 mM d-Glucose, HEPES 10 mM, pH was set to 7.4 using 1.5 M Tris in MQ, 37 °C) HT-buffer containing 100 µM ASP^+^ was added and the cells were incubated for 15 min at 37 °C. Intracellular uptake of the fluorescent compound (dimethylamino)styryl)-N-methylpyridinium-iodide (ASP^+^) was measured using a Zeiss Apotome Fluorescence microscope. Images were recorded over a time period of 15 min, starting 2 min after addition of 10 µM ASP^+^ to enable a good focus on the cellular monolayer (Zeiss Axiovision imaging software 4.7.2). Fluorescence intensity over time was quantified for at least 6 individual cells for each condition, by plotting the Z-axis profile of the virtual stack using ImageJ software (ImageJ 1.46r, NIH, Bethesda, MA, USA).

To enable high throughput evaluation of organic cation uptake following stimulation by ET-1, in combination with pharmacological modulation of the signaling pathway, a fluorescence reader based method was used. Matured cells, cultured in 12 wells plates (seeded at approx. 150,000 cells/well) were treated with fresh medium containing the test-compounds, inhibitor or control medium for the designated incubation period. Subsequently, the cells were washed with Hepes Tris buffer (pH 7.4). HT-buffer containing 10 µM ASP^+^ was added and the cells were incubated for 15 min at 37 °C. Next, the uptake was arrested by washing twice with ice-cold stop solution (0.5mM TPA in HT-buffer). Then, the cells were lysed for 30 min lysis buffer (0.05% w/v Saponin, 0.05% v/v triton in MilliQ) and the cell homogenate transferred to a 96 wells plate (Greiner). The fluorescence measurement was performed three times at 450–642nm VictorX multilabel plate reader (PerkinElmer, Waltham, MA, USA).

### 2.6. Data Analysis

Values are given as mean ± standard error of the mean. Fluorescence levels are normalized to the unexposed control samples after subtraction of the background fluorescence at baseline (t = 0). Mean values were considered to be significantly different when *p* < 0.05 using a one-way ANOVA followed by Dunnett’s multiple comparison test. Software used for statistical analysis was GraphPad Prism (version 5.00 for Windows; GraphPad Software, San Diego, CA, USA).

## 3. Results and Discussion 

### 3.1. Various Cationic Uremic Toxins Induce IL-6, IL-8, TNFα and ET-1 Production by ciPTEC

**Figure 1:**
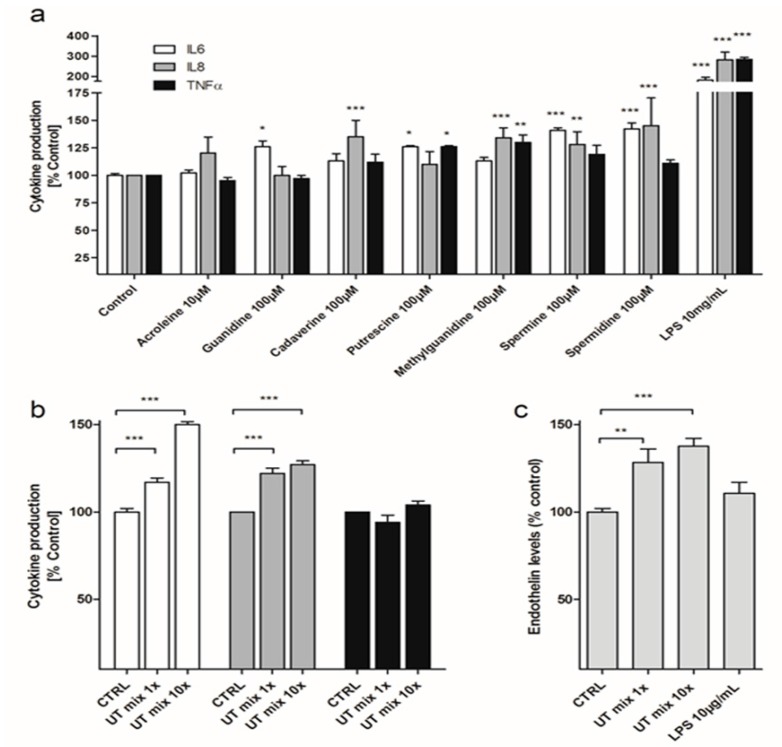
TNFα, IL-6, IL-8 and ET-1 excretions by ciPTEC after exposure to cationic UTs. (**a**) TNFα, IL-6 and IL-8 excretion was measured 24 h after incubation of ciPTEC with 10 µM acrolein, 100 µM of guanidine, cadaverine, putescine, methylguanidine, spermine, spermidine, or 10 µg∙mL^−1^ LPS; (**b**) TNFα, IL-6 and IL-8 excretion was measured 24 h after incubation of ciPTEC with a mixture of above stated cationic UTs, corresponding to one-fold or 10-fold the levels reported in uremic patients; (**c**) ET-1 production by ciPTEC after 24 h of incubation with the mixture of cationic UTs or 10 µg/mL LPS. Data are expressed as the percentage of cytokines or ET-1 produced compared with untreated cells, set at 100%. Results are shown as normalized means ± SEM. For each condition at least three experiments were performed in triplicate (^*^ *p* < 0.05, ^**^ *p* < 0.01, ^***^ *p* < 0.001, ANOVA).

Production of the pro-inflammatory cytokines IL-6, IL-8 and TNFα by ciPTEC was measured in culture supernatant after exposure to a selection of cationic UTs (Figure 1a), which have previously been shown to interfere with tubular organic cation uptake. To assess the potencies of the individual UTs on cytokine production, ciPTEC were exposed to 100 µM of guanidine, cadaverine, putrescine, methylguanidine, spermine, spermidine or 10 µM of acrolein for 24 h. Acrolein was used at a lower concentration to prevent severe cytotoxicity [2]. Average baseline IL-6, IL-8 and TNF-α production by ciPTEC over 24 h were determined to be 51 ± 5.4 ng∙mL^−1^, 79 ± 37 ng∙mL^−1^ and 227 ± 35 pg∙mL^−1^ respectively. Compared to controls, IL-6 production increased significantly after stimulation by guanidine (126 ± 5%, *p* < 0.05), putrescine (126 ± 1%, *p* < 0.05), spermine (141 ± 2%, *p* < 0.001) and spermidine (142 ± 6%, *p* < 0.001). IL-8 production was stimulated by 100 µM of cadaverine (135 ± 15%, *p* < 0.01), methylguanidine (134 ± 9%, *p* < 0.001) spermine (128 ± 12%, *p* < 0.01) and spermidine (145 ± 25%, *p* < 0.001). We also detected a significant rise in TNFα production after exposure to 100 µM of putrescine (126 ± 1%, *p* < 0.05) and methylguanidine (130 ± 7%, *p* < 0.05; Figure 1a). Lipopolysaccharide (LPS, 10 µg∙mL^−1^) was used as a positive control, which caused a significant increase in IL-6 (181 ± 14%, *p* < 0.001), IL-8 (282 ± 23%, *p* < 0.001) and TNFα levels (284 ± 9%, *p* < 0.001).

When ciPTEC were exposed to a mixture of the selected cationic UTs (*i.e.*, toxin concentrations corresponding to those found in uremic patients, see Methods Section for actual concentrations used; Figure 1b) for 24 h, a significant increase in IL-6 (117 ± 3%, *p* < 0.001) and IL-8 (122 ± 3%, *p* < 0.001) cytokine levels could be detected. When increasing the UT mixture to 10-fold the concentrations reported in patients, IL-6 and IL-8 levels increased even further, until 150 ± 2% and 127 ± 2% (*p* < 0.001) when compared to control cells, respectively. TNFα levels did not rise upon exposure to these uremic mixtures.

Production of the vasoactive peptide and inflammatory mediator ET-1 by ciPTEC was measured in culture supernatant after exposure to the mixture of the selected cationic UTs for 24 h. Figure 1c depicts the ET-1 release by ciPTEC. Average baseline ET-1 production by ciPTEC over 24 h was determined to be 14.6 ± 4 pg∙mL^−1^ (11 ± 4 pg∙mg^−1^protein). Exposure to the one-fold and 10-fold cationic UT mixture for 24 h increased ET-1 production by ciPTEC, up to 128 ± 7% (*p* < 0.01) and 134.4 ± 5% (*p* < 0.001), respectively. Exposure of ciPTEC to the individual cationic uremic compounds for 24 h, did not lead to significantly affected ET-1 release (data not shown). Remarkably, LPS did not induce ET-1 production by ciPTEC.

### 3.2. Differential Effect of ET-1 Exposures on Organic Cation Transport

**Figure 2:**
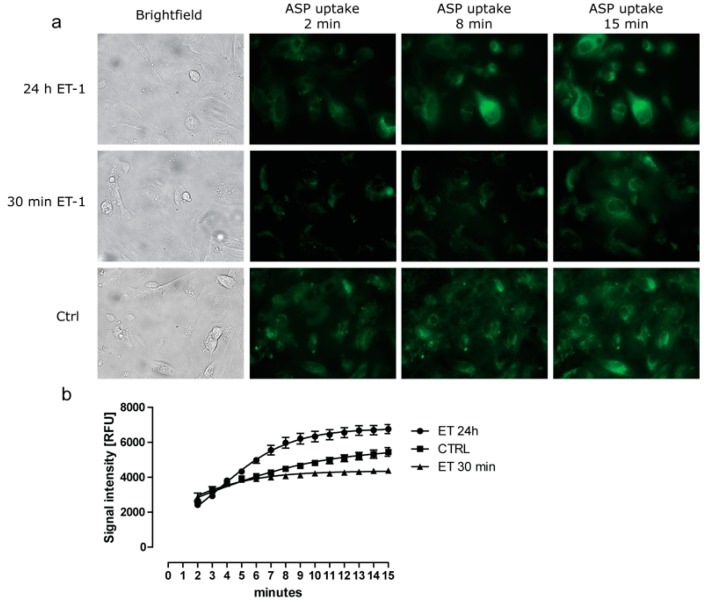
Changes in ASP^+^ uptake by ciPTEC in response to exposure to ET-1. CiPTEC were incubated for 30 min or 24 h with 100 nM ET-1 before measuring the intracellular uptake of the fluorescent cationic compound ASP^+^ (10 µM). (**a**) Representative confocal images of 24 h ET-1 exposed (top row), 30 min ET-1 exposed (middle row) or control cells (bottom row) taken at 2, 8 or 15 min after addition of ASP^+^; (**b**) ASP^+^ accumulation in cells was determined over the 15 min time period for 24 h ET-1 exposed (●), 30 min ET-1 exposed (▲) or control cells (■). Fluorescence intensity over time was quantified for at least six individual cells for each condition. Images were captured by real time imaging. Prior to addition of the fluorescent compound, brightfield images (panel A) were taken. The addition of the fluorescent substrate resulted in minor cell movement, thereby affecting cell positioning to some extent.

To investigate if ET-1 could affect the functionality of the organic cation transporters (OCT) present in ciPTEC, we measured the intracellular uptake of the fluorescent compound 4-(4-ASP^+^ after exposure to ET-1. A dose-response curve showed a significant increase in ASP^+^ uptake at concentrations of 100 nM and 1 µM ET-1 (Figure S1). As a control, TPA was used which inhibited ASP uptake by approx. 70%. In Figure 2 representative fluorescence images (A) and their quantified fluorescent signals (B) are shown for the ASP^+^ uptake by ciPTEC in the presence or absence of 100 nM ET-1 exposure. A significant increase in maximal uptake was observed after 24 h of incubation, while 30 min of exposure diminished the uptake compared to unexposed cells.

**Figure 3:**
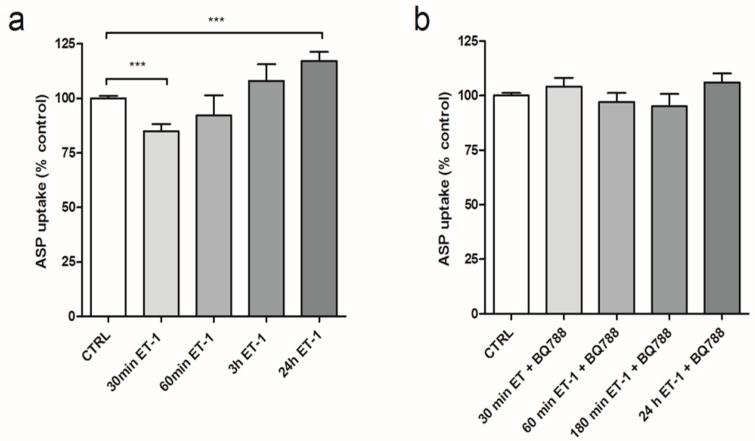
Regulatory effects on OCT mediated ASP^+^ uptake through ET-1 Receptor. Pre-incubation of ciPTEC with 100 µM ET-1 for 30 min up to 24 h inhibits the cellular uptake of 10µM of the fluorescent substrate ASP^+^. (**a**) The effects of 100 μM ET-1 on steady state (15 min) ASP^+^ accumulation; (**b**) ASP^+^ accumulation of ciPTEC after pre-incubation with a combination of 100 nM ET-1 with the ET-B receptor inhibitor BQ788 (1 µM), which blocked the effect of ET-1 at all time points. Values are depicted as means ± SEM for at least three experiments performed in triplicate (^*^ *p* < 0.05, ^**^ *p* < 0.01, ^***^ *p* < 0.001).

By using a fluorescent reader based method, more conditions were tested in a high throughput fashion (Figure 3a). Again, after 30 min of pre-incubation with 100 nM ET-1, a 23 ± 4% (*p* < 0.001) decrease in ASP^+^ uptake was measured. This effect was not observed after increasing the pre-incubation time to 3 or 6 h. However, after 24 h of pre-incubation, a 20 ± 5% (*p* < 0.001) increase in ASP^+^ uptake was observed. Addition of the ET-1 receptor inhibitor BQ-788 during the exposures normalized the ASP^+^ uptake levels for all time points (Figure 3b).

In humans, OCT2 (SLC22A2) is considered one of the most important renal OCT [33]. We examined its mRNA expression levels after exposure to 100 nM ET-1 for 30 min or 24 h and compared these results to the control situation. Additionally, the expression levels of OCT1 and OCT3 were examined as these are expressed in ciPTEC as well [2,34]. Exposure to ET-1 did not significantly alter OCT1, 2 or 3 expression levels for short or long term (data included as Figure S2).

### 3.3. iNOS Inhibition Attenuates the Inhibitory Effects of ET-1 Exposure on Organic Cation Uptake

**Figure 4:**
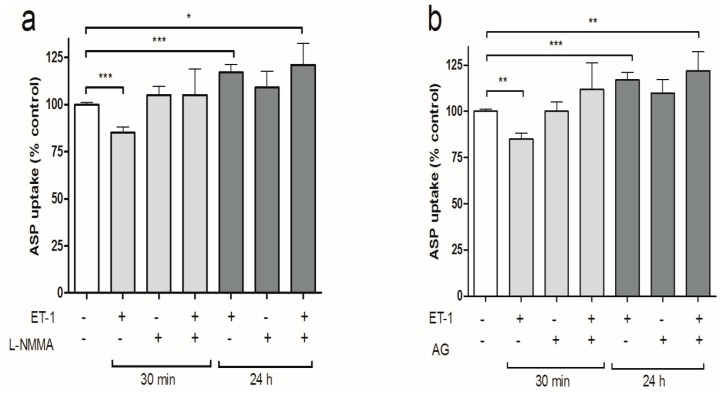
NOS-inhibition reverses the short-term action of ET-1 on ASP^+^ uptake. CiPTEC monolayers were incubated for 15 min in medium containing 10 µM ASP^+^ without or with pre-incubation with (**a**) 100 nM ET-1, 100 μM L-NMMA or ET-1 plus L-NMMA; (**b**) 100 nM ET-1, 100 μM aminoguanidine (AG) or ET-1 plus 100 µM AG. Subsequently, the reaction was stopped, the cells were lysed and the fluorescent signal was measured as described in the methods section. Values are presented as means ± SEM for at least three experiments performed in triplicate. (^*^ *p* < 0.05, ^**^ *p* < 0.01, ^***^ *p* < 0.001).

Previous studies demonstrated that ET-B receptor activation can initiate an NO mediated signaling pathway that regulates drug transport in the proximal tubule [24]. Therefore, we investigated if the differential regulation of intracellular organic cation uptake induced by ET-1, resulted from nitric oxide synthase (NOS) activation. ciPTEC were pre-incubated with 100 nM ET-1 in the absence or presence of the nonselective NOS‐inhibitor l‐arginine analogue N(G)‐monomethyl‐l‐arginine (l-NMMA). Figure 4a shows that l-NMMA alone did not influence ASP^+^ uptake by ciPTEC, but attenuated the ET-1 mediated down-regulation in ASP^+^ uptake at 30 min. This effect was not observed after the 24 h pre-incubation period. Pre-incubation with the selective inducible NOS inhibitor AG in the presence or absence of ET-1 produced similar results, attenuating only the short-term ET-1 mediated effect (Figure 4b).

### 3.4. PKC Activation Restores Short-Term Organic Cation Uptake in ciPTEC after ET-1 Exposure

**Figure 5:**
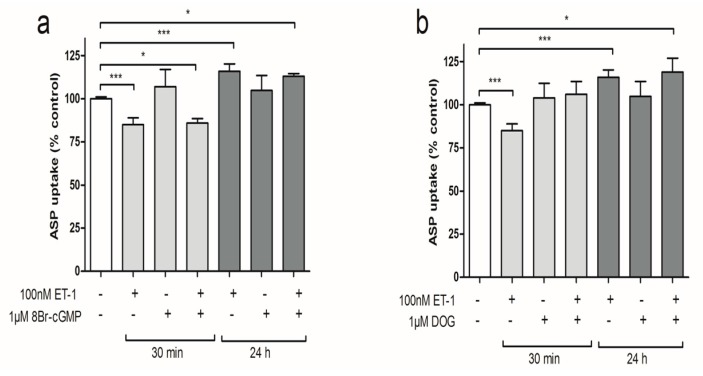
Kinase pathways on ciPTEC ASP+ uptake levels after exposure to ET-1. ciPTEC monolayers were incubated for 15 min in medium containing 10 µM ASP^+^ without or with pre-incubation with (**a**) 100 nM ET-1, 1 μM DOG or ET-1 plus DOG; (**b**) 100 nM ET-1, 1 μM 8-br-cGMP or ET-1 plus 100µM 8-br-cGMP. Subsequently, the reaction was stopped, the cells lysed and the fluorescent signal was measured as described in the methods section. Values are expressed as means ± SEM for at least three experiments performed in triplicate. (^*^ *p* < 0.05, ^**^ *p* < 0.01, ^***^ *p* < 0.001).

Cyclic guanosine monophosphate (cGMP) activation was simulated by the addition of 1 µM of 8-br-cGMP, a cell permeable analog of cGMP, which was previously identified as an important signaling molecule in ET-1 regulated MRP2 transport in the proximal tubule [35]. After pre-incubation of ciPTEC with 1 µM 8-Br-cGMP alone, the intracellular ASP^+^ levels did not differ from the control conditions (Figure 5a). Also, combining the pre-incubation to ET-1 exposure with 8-Br-cGMP could not prevent either the inhibitory effect on ASP^+^ uptake at 30 min, or the stimulation observed after 24 h. These results point towards an absence of protein kinase G and cGMP as signaling molecule in this ET-1-mediated pathway. Protein kinase C was stimulated by pre-incubation with the PKC activator sn-1,2-dioctanoyl glycerol (DOG), which is a cell permeable analog of the PKC-activating second messenger diacylglycerol (DAG). Pre-incubation of ciPTEC with 1 µM DOG alone did not alter ASP^+^ uptake compared to the non-exposed control situation (Figure 5b). Combining ET-1 and DOG during 30 min of pre-incubation restored the ASP^+^ uptake by ciPTEC to the control situation. This effect of DOG was not observed after 24 h of ET-1 incubation, suggesting that two separate pathways regulate organic cation transport.

### 3.5. Discussion

The results of the present study indicate that exposure of human proximal tubule epithelial cells to cationic UTs leads to cytokine and ET-1 production by ciPTEC. We demonstrated that the production of IL-6, IL-8, and TNFα was stimulated by the exposure to high levels of individual cationic toxins, supporting the inflammatory response often observed in uremia. At clinically relevant concentrations, a mixture of these selected toxins induced the endogenous production of IL-6, IL-8 and ET-1. Additionally, ET-1 was identified as time-dependent regulator of organic cation uptake through interaction with the ET-B receptor.

In patients suffering from renal failure, the accumulation of UTs was reported to be associated with the development of a chronic inflammatory state [9]. Various clinical studies have demonstrated a correlation between increasing IL-6, IL-8 and TNFα cytokine levels and reduced glomerular filtration rate [11,12]. Inefficient clearance of the inflammatory mediators in combination with increased cytokine production could cause these effects. The results of the present study confirm that exposure to a cationic uremic mixture of acrolein, spermine, spermidine, cadaverine, putrescine, guanidine and methylguanidine induced the production of IL-6 and IL-8 by using the human renal cell line ciPTEC. 

The observed increase in IL-6 and IL-8 production ranged from 26%–45%. When compared to the 81%–182% increase in IL-6 and IL-8 release after LPS stimulation, this level could be considered modest. However, LPS is one of the most potent inducers of cytokine production. Patients suffering from ESRD were reported to show a 35% rise in IL-6 levels [11]. The local production of these cytokines in response to a uremic milieu can promote local profibrotic or inflammatory processes *in-vivo*, since IL-6 production is associated with the induction of fibrotic gene expression, and IL-8 is a potent neutrophil chemoattractant [36,37]. Therefore, we could consider the 26%–45% increase in production of IL-6 and IL-8 by proximal tubule epithelial cells clinically relevant. It is, however, important to take into account that our *in vitro* settings cannot be translated directly to the clinical situation, especially with regard to protein binding and intracellular solute concentrations. Not only are organic solutes often able to bind to human serum albumin, they can also interact with other proteins such as α1-acid glycoprotein and lipoproteins, and even red blood cells and platelets [38]. On the other hand, it is known that ESRD can affect systemic protein binding of drugs and endogenous compounds due to displacement [39,40,41]. Protein binding could, therefore, affect the free fraction of uremic solutes available to cells. Nevertheless, intracellular levels often easily exceed the free serum concentrations due to active uptake processes [42,43].

The absence of TNFα production by ciPTEC after exposure to the UT mixture contrasts with the effects of the individual compounds putrescine and methylguanidine. In the mixture, the concentrations of these compounds were much lower as compared to the experiments performed with the individual compounds, which might explain this effect. Additionally, TNFα is known to possess a very short half-life in whole blood [44], and low concentrations are reported to denature rapidly in culture medium at 37 °C [45,46]. Since ciPTEC were exposed for 24 h, the initial TNFα peak could have subsided before the measurement was performed.

Many cytokines, including IL-6, are known to stimulate ET-1 production. Furthermore, the proximal tubule can endogenously produce ET-1 upon stimulation with various nephrotoxicants, as was previously demonstrated in non-human species [24,47,48]. Here we show that, next to the production of IL-6 and IL-8, the mixture of cationic UTs also promoted a modest ET-1 release by ciPTEC. The local production of ET-1 in the renal tubule has been recognized as an early response to tubular injury, and was identified as a key regulator of efflux transporters, MRP2 and P-gp [23,26,35,47,48,49]. In these studies, the threshold for ET-1 action on transporters was determined to be between 0.5 and 10 nM (1.25–24.9 ng∙mL^−1^). In our study, the ET-1 release by ciPTEC was well below this threshold (Figure 1). The quantity of hormone released by ciPTEC is small, and much diluted by the cell culture medium. However, since the cells produce this compound endogenously, the threshold level could be reached in the direct vicinity of the cells.

The results of the present study also demonstrate that next to the regulation of MRP2 and P-gp, ET-1 can differentially regulate cation uptake in ciPTEC. Because ET-1 is a hydrophilic compound, surface receptors are necessary to regulate intracellular responses to the peptide. The regulatory effects of ET-1 on the OCT-mediated ASP^+^ uptake were clearly mediated through the action of the ET-B-receptor, since the addition of the ET-B-receptor blocker BQ-788 ameliorated both effects observed after 30 min and 24 h.

The inhibition of OCT-mediated ASP^+^ transport by ET-1 after 30 min is in line with earlier reports. Terlouw *et al.* previously hypothesized that the reduction in efflux activity by transporters could protect the cell form injury after exposure to a nephrotoxic substance by saving ATP for more immediate processes that are necessary for cell survival [24,48]. Although the reduction in ASP^+^ uptake by ciPTEC after 30 min suggests a similar protective mechanism for OCT, the reduced influx is unlikely to serve as an ATP saving mechanism. Organic cation uptake processes are mostly mediated by polyspecific solute carriers like OCT2 (*SLC-22* family), which are membrane potential and substrate concentration gradient dependent, and therefore function as facilitated diffusion carriers for organic cations, but are independent of proton gradients. In addition, two other types of apically expressed transporters might be involved, *viz.* the Carnitine/Organic Cation Transporters OCTN1 and OCTN2 (SLC22A4 and SLC22A5 [50,51]) and the proton antiporters, MATE1 and MATE2k (multi-antimicrobial extrusion proteins). Schmidt-Lauber *et al.* reported active uptake of ASP^+^ through OCTN1 and MATE1 in transporter-transfected HEK293 cells and human synovial fibroblasts (hRASF) [52]. In ciPTEC, the exact role of these transport proteins in the uptake of cationic organic substrates, such as ASP^+^ or uremic retention solutes, is yet unclear. Future studies should be directed to further elucidate their role.

Solute carriers do not rely directly on ATP binding or hydrolysis [53,54]. Based on our results, we suggest that the short-term reduction in organic cation influx by ET-1 signaling provides protection by minimizing intracellular accumulation of potentially toxic compounds. In acute situations, this would reduce intracellular damage until other clearance processes are activated to handle the toxic threat. On the other hand, the increased influx transport observed after 24 h was unexpected. This observation could not be linked to increased mRNA levels of OCT2 neither OCT1 or OCT3, of which detection on mRNA level in ciPTEC was remarkable, since they are generally considered of less importance in human kidney [33,34] (Figure S2). We cannot exclude the involvement of another important mechanism in the short-term regulation of transporter activity known as endocytic membrane retrieval, or insertion as suggested previously [55,56]. This mechanism has also been described in the short-term regulation of the proximal tubular type IIa Na-P_i_ cotransporter, and the pH-regulated insertion of H^+^-ATPases in the proximal tubule [57,58,59,60]. Furthermore, rapid insertion of MRP2 in response to tubulo toxic insults has been described [26,61]. If OCT functionality is regulated by such rapid dynamic endocytic retrieval or insertion processes, the regulation depends on an intracellular vesicular pool of transporters, and its regulation will therefore not directly affect mRNA expression levels. The development of specific antibodies directed at the human organic cation transporters, together with immunocytochemistry or protein expression studies, could in the future provide more insight into the involvement of the regulatory mechanisms of organic cation uptake in ciPTEC. Furthermore, post-translational modifications, such as phosphorylation, could affect transporter activity. In the intracellular loops of the (human) *SLC-22* family, several potential phosphorylation sites have been identified [62]. For rOCT1, phosphorylation events are known to stimulate conformational changes at the substrate binding site, thereby increasing the affinity for its substrates [63]. In various studies focusing on ASP^+^ uptake in rabbit or human models, PKC stimulation was found to either induce or inhibit substrate uptake, depending on the species [64].

In ciPTEC, PKC stimulation by DOG in combination with ET-1 restored cation uptake after 30 min of pre-incubation. These observations suggest that PKC activation in ciPTEC stimulates organic cation uptake, however, this is in contrast to previous studies with hOCT2-HEK293 cells and isolated human proximal tubules [55,64]. The reason for this discrepancy is unclear, but considering the many intracellular processes affected by PKC this may suggest that multiple regulatory pathways are involved. Still, the findings of the present study are in agreement with the known interaction between ET-1 and the protein kinase pathway in the regulation of other renal transporters (*viz.* MRP2 and P-gp) [23,65,66]. In killifish renal proximal tubules, dogfish shark salt glands and rat brain capillaries, ET-1 also reduced MRP2 or P-gp-mediated transport by stimulating the PKC pathway [23,65,66]. It would therefore be interesting to evaluate in future studies how ET-1 production by ciPTEC would affect the transport by MRP2 or P-gp in this cell model.

Next to the PKC pathway, our results demonstrated that inhibition of iNOS by AG or L-NMMA restored ASP^+^ uptake at 30 min after ET-1 exposure. In the human proximal tubule, iNOS is constitutively expressed [67,68]. It mediates the regulation of local inflammatory responses, following cytokine production during endotoxemia, or after exposure to nephrotoxicants [47,48]. Similar to the iNOS induced effects observed in the present study, Heemskerk *et al.* demonstrated a reduction in OCT1 and OCT2 uptake transport during acute endotoxemia, while the functional expression of the efflux transporters MRP2 and P-gp, increased [25,69]. Therefore, iNOS is considered an important player in the regulation of the short-term tubular response to external assaults. These short-term effects of the inflammatory mediator ET-1 on the regulation proximal tubular cation uptake could provide clues on possible pathways involved in acute kidney injury.

Though numerous studies have investigated the mechanisms involved in renal proximal tubular transport, the mechanisms behind these regulatory pathways is often analyzed within relatively short time frames. We observed a time-dependent ET-B receptor mediated effect of ciPTEC exposure to ET-1, leading to an increased cellular uptake of cationic substances. The mechanisms behind this long-term (24 h) regulation could not be explained by the known NO, PKC or cGMP mediated pathways. These observations are reminiscent of both the biphasic and NO-independent pathways that were described for the efflux transporter P-gp [70,71], for which a second pathway, next to NO mediated regulation, was identified involving activation of Toll Like Receptor 4 and translocation of NF-κB. Further research would be warranted to investigate this pathway in the regulation of proximal tubular organic cation uptake. Investigating the increase in substrate uptake following long term ET-1 exposure might provide clues about possible treatment strategies to promote renal secretory clearance in uremia, or shed further light on protective mechanisms of the renal proximal tubule.

In conclusion, our findings implicate that cationic UTs can directly induce the local production of inflammatory cytokines and ET-1. Furthermore, ET-1 exposure was found to enable regulation of organic cation uptake by proximal tubule cells. These findings might suggest that systemic accumulation of UTs in patients suffering from CKD could influence renal tubular clearance processes through local production of inflammatory or vasoactive mediators. However, further studies on OCT regulation and functionality in response to UT exposure are required to elucidate the exact processes that connect these events.

## Supplementary Materials

Supplementary materials can be accessed at: http://www.mdpi.com/2073-4409/4/3/234/s1.
