# Supplementary material for: Uremic Toxins Induce ET-1 Release by Human Proximal Tubule Cells, which Regulates Organic Cation Uptake Time-Dependently
Source: Cells. 2015 Jun 26;4(3):234–52. doi: 10.3390/cells4030234 (PMC4588034; doi:10.3390/cells4030234)
Supplement: Supplementary file 1 [file cells-04-00234-s001.pdf]

Supplementary Materials

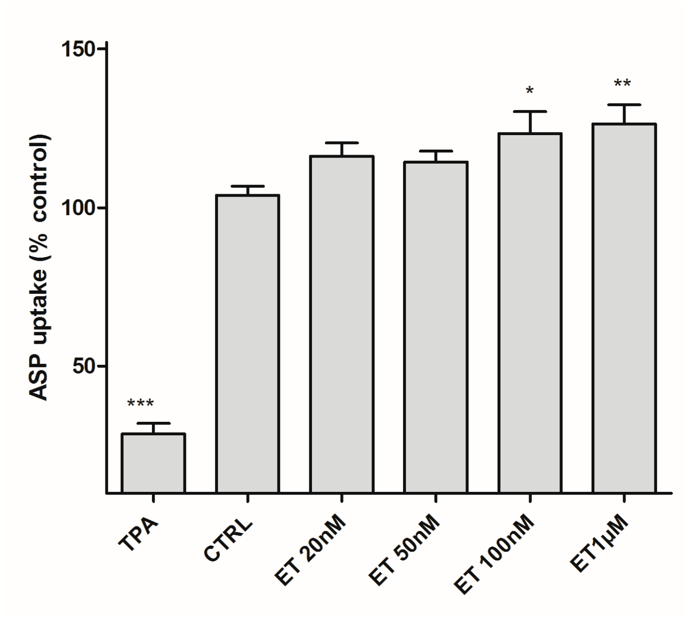

Figure S1. ciPTEC ASP$^+$ uptake levels, after exposure to a dose-range of ET-1 for 24 hours. CiPTEC monolayers were incubated for 15 min in medium containing 10 µM ASP+ without or with pre-incubation with 0-1µM ET-1, for 24 hours. As a control, the OCT inhibitor 100 µM TPA was used which inhibited ASP+ uptake by approx. 70%. Subsequently, the reaction was stopped, the cells were lysed and the fluorescent signal was measured as described in the methods section. Values are expressed as mean ± SEM for 2 experiments performed in triplicate. (* $p < 0.05$, ** $p < 0.01$, *** $p < 0.001$).

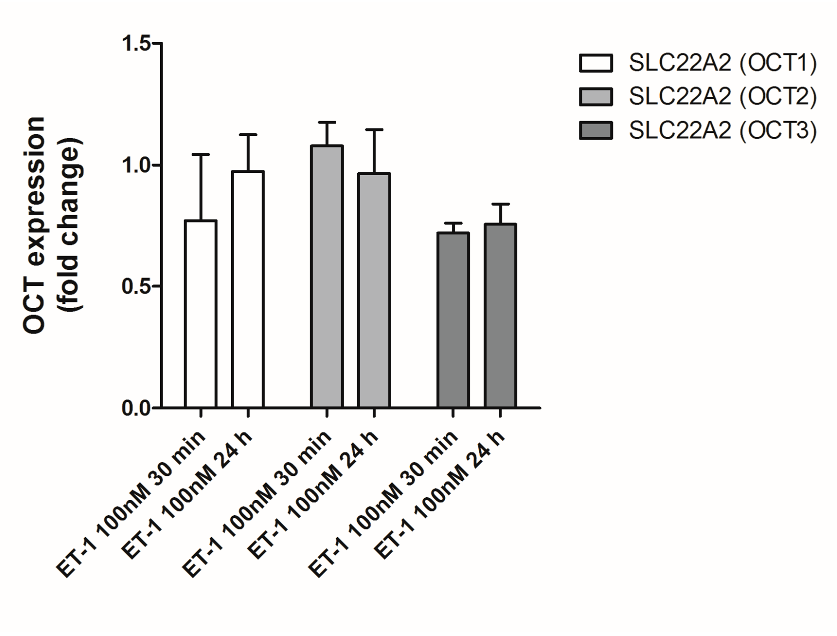

Figure S2. ET-1 does not significantly change OCT expression on mRNA level. Fold changes in mRNA expression levels of OCT2 corrected for corresponding GAPDH mRNA levels expressed as fold increase compared to non- ET-1 exposed ciPTEC (Control ciPTEC Δc(t) values OCT1: 15 ± 0.7, OCT2: 18 ± 0.52, OCT3:8 ± 0.5)
